# Cardiopulmonary Exercise Performance and Endothelial Function in Convalescent COVID-19 Patients

**DOI:** 10.3390/jcm11051452

**Published:** 2022-03-07

**Authors:** Pasquale Ambrosino, Paolo Parrella, Roberto Formisano, Giovanni Perrotta, Silvestro Ennio D’Anna, Marco Mosella, Antimo Papa, Mauro Maniscalco

**Affiliations:** 1 Istituti Clinici Scientifici Maugeri IRCCS, Cardiac Rehabilitation Unit of Telese Terme Institute, 82037 Telese Terme, Italy; roberto.formisano@icsmaugeri.it (R.F.); giovanni.perrotta@icsmaugeri.it (G.P.); antimo.papa@icsmaugeri.it (A.P.); 2 Ospedale Sacro Cuore di Gesù Fatebenefratelli, 82100 Benevento, Italy; paoloparrella01@libero.it; 3 Istituti Clinici Scientifici Maugeri IRCCS, Pulmonary Rehabilitation Unit of Telese Terme Institute, 82037 Telese Terme, Italy; silvestro.danna@icsmaugeri.it (S.E.D.); marco.mosella@icsmaugeri.it (M.M.)

**Keywords:** COVID-19, SARS-CoV-2, endothelial function, chronic disease, cardiovascular diseases, disability, exercise, rehabilitation, occupational medicine, outcome

## Abstract

Background: Endothelial dysfunction has been proposed as the common pathogenic background of most manifestations of coronavirus disease 2019 (COVID-19). Among these, some authors also reported an impaired exercise response during cardiopulmonary exercise testing (CPET). We aimed to explore the potential association between endothelial dysfunction and the reduced CPET performance in COVID-19 survivors. Methods: 36 consecutive COVID-19 survivors underwent symptom-limited incremental CPET and assessment of endothelium-dependent flow-mediate dilation (FMD) according to standardized protocols. Results: A significantly higher FMD was documented in patients with a preserved, as compared to those with a reduced, exercise capacity (4.11% ± 2.08 vs. 2.54% ± 1.85, *p* = 0.048), confirmed in a multivariate analysis (β = 0.899, *p* = 0.038). In the overall study population, FMD values showed a significant Pearson’s correlation with two primary CPET parameters, namely ventilation/carbon dioxide production (VE/VCO_2_) slope (r = −0.371, *p* = 0.026) and end-tidal carbon dioxide tension (P_ET_CO_2_) at peak (r = 0.439, *p* = 0.007). In multiple linear regressions, FMD was the only independent predictor of VE/VCO_2_ slope (β = −1.308, *p* = 0.029) and peak P_ET_CO_2_ values (β = 0.779, *p* = 0.021). Accordingly, when stratifying our study population based on their ventilatory efficiency, patients with a ventilatory class III-IV (VE/VCO_2_ slope ≥ 36) exhibited significantly lower FMD values as compared to those with a ventilatory class I-II. Conclusions: The alteration of endothelial barrier properties in systemic and pulmonary circulation may represent a key pathogenic mechanism of the reduced CPET performance in COVID-19 survivors. Personalized pharmacological and rehabilitation strategies targeting endothelial function may represent an attractive therapeutic option.

## 1. Introduction

Despite the different phenotypic characteristics displayed by endothelial cells (ECs) in different organs and tissues, endothelial dysfunction shares some common features, such as reduced vasodilation, inflammation, oxidative stress and a prothrombotic state. Thus, the presence of a dysfunctional endothelium has been proposed as a key and early pathogenic mechanism in many clinical conditions [1].

Accumulated evidence has suggested that endothelial dysfunction may be the common pathogenic background of most manifestations of coronavirus disease 2019 (COVID-19), since ECs are a preferential target of the novel severe acute respiratory syndrome coronavirus 2 (SARS-CoV-2) [2]. This has led the European Society of Cardiology (ESC) to recommend the clinical assessment of endothelial function in the follow-up of all convalescent COVID-19 patients, aimed at monitoring the risk of long-term cardiovascular complications [3]. For this purpose, several methods have been proposed, including measurement of flow-mediated dilation (FMD), which is widely accepted as one of the most reliable and cost-effective procedures for evaluating endothelial function [4].

Among a number of clinical manifestations related to SARS-CoV-2 infection, some authors also reported an impaired exercise response in COVID-19 survivors during cardiopulmonary exercise testing (CPET) [5,6], which is the gold standard for assessing exercise capacity. Thus, the European Respiratory Society/American Thoracic Society (ERS/ATS) task force has also recommended CPET in the follow-up of COVID-19 [7].

Given the key role of ECs integrity in maintaining homeostasis of the cardiovascular and respiratory systems [1], we aimed to explore the potential association between endothelial dysfunction and the reduced cardiopulmonary exercise performance in convalescent COVID-19 patients.

## 2. Materials and Methods

### 2.1. Patients

From December 2020 to April 2021, convalescent COVID-19 patients admitted to the Pulmonary Rehabilitation Unit of Istituti Clinici Scientifici Maugeri IRCCS, Telese Terme, Benevento, Italy were consecutively screened for inclusion within 2 months from swab test negativization, according to the following criteria: age ≥ 18 years; severe-to-critical COVID-19 [8], confirmed by reverse transcription polymerase chain reaction (RT-PCR); computed tomography (CT) evidence of recent interstitial pneumonia (e.g., ground glass opacities, crazy paving); at least 2 negative swab tests for SARS-CoV-2 (spaced 1 week apart) in the past 2 months; clinical conditions sufficient to initiate an exercise-based rehabilitation program. Exclusion criteria were: active malignancy; history of lung surgery; any major surgery within the last 6 months; history of cardiovascular or respiratory disease, including stroke, transient ischemic attack, myocardial infarction, atrial fibrillation, heart failure, peripheral artery disease, chronic obstructive pulmonary disease, asthma; history of interstitial lung disease of different origin (autoimmune, genetic, idiopathic, exposure to hazardous materials) or CT evidence of chronic pre-existing lung fibrosis (e.g., honeycomb lung, traction bronchiectasis); history of chronic kidney disease; previous participation in any rehabilitation program following swab test negativization. Wherever appropriate and applicable, this study was reported following the Strengthening the Reporting of Observational Studies in Epidemiology (STROBE) reporting guidelines [9]. The protocol was approved by a competent ethics committee (reference number ICS-11/20), in line with the principles of the 1975 Helsinki Declaration.

### 2.2. Study Procedures

After informed consent signature, the major demographic and clinical characteristics were collected. Moreover, the main anthropometric, laboratory, echocardiographic and pulmonary function parameters were measured in each patient, following standardized protocols [10,11,12]. An expert operator (P.A.) assessed parameters of vascular reactivity in real time using an automatic edge detection software (Cardiovascular Suite^®^, FMD studio, QUIPU Srl, Pisa, Italy), cleared by the Food and Drug Administration (FDA). Briefly, participants were asked to abstain from tobacco, food, and caffeine for 12 h prior to the exam. After 10 min of supine rest, the patient kept his right arm abducted 90 degrees in the frontal plane, with a pressure cuff placed on the forearm. Then, brachial artery diameter (BAD) and blood flow velocity were monitored for 10 min with an ultrasound equipment before, during and after 5 min cuff inflation to a supramaximal pressure [13]. FMD, representing the percent change in BAD after cuff deflation, and other vascular reactivity parameters were automatically calculated. Overall, the main study procedures have been detailed elsewhere [14].

In addition, according to standardized protocols [15], all included patients underwent symptom-limited incremental CPET until maximum exhaustion using an electronical cycle ergometer for gradually increasing workload and a JAEGER^®^ Vyntus CPX (Jaeger-CareFusion, Hoechberg, Germany) equipment for measuring gas exchange and ventilation. In line with clinical recommendations for CPET in specific patient populations [16], the absolute value of peak oxygen uptake (VO_2_) was used as a primary CPET variable. Thus, according to the Weber classification [17], patients were considered to have a reduced exercise capacity if peak VO_2_ was less than 20 mL/kg/min.

### 2.3. Statistical Analyses

Statistical analyses were carried out with the IBM SPSS Statistics 28.0 system (Chicago, IL, USA). Continuous data were expressed as mean ± standard deviation or median (1st–3rd quartile) in case of skewed distribution. Categorical variables were summarized as relative frequencies. Student’s *t* test was computed for normally distributed quantitative variables and the Mann–Whitney *U* test for non-normal quantitative and ordinal variables. Pearson’s chi-squared test with Yates continuity correction was used to compare dichotomous variables. Pearson or Spearman correlation coefficients were used to examine the relationship between continuous variables. Linear and binary logistic regression analyses were used to adjust for any potential confounder (age, body mass index, hypertension, dyslipidemia, diabetes, smoking habit) and to identify predictors. A *p* value < 0.05 (2-sided) was considered statistically significant. 

## 3. Results

As shown in Supplemental Figure S1, given the exclusion of patients with any history of cardiovascular or pulmonary disease potentially impacting exercise performance and vascular reactivity, among 112 convalescent COVID-19 patients screened for eligibility, 57 (50.9%) were ineligible for protocol adherence issues. A total of four (7.3%) out of the 55 eligible patients dropped out before completion of the project requirements, while 15 (27.3%) were not considered due to inability to complete CPET or unsuccessful FMD measurement. Thus, a case series of 36 participants (91.7% males, mean age 54.5 years) was included in the final analysis (Table 1).

All patients successfully completed the CPET procedures, given that a respiratory exchange ratio of 1.05 was achieved [18]. The mean ± standard deviation peak VO_2_ value in our study population was 16.6 ± 3.9 mL/min/kg, corresponding to a 62.4 ± 16.1% of predicted, with a ventilation/carbon dioxide production (VE/VCO_2_) slope of 35.5 ± 5.3 and end-tidal carbon dioxide tension (P_ET_CO_2_) at peak of 32.3 ± 3.2 mmHg. A total of eight patients (22.2%) exhibited a peak VO_2_ > 20 mL/kg/min, being not different in demographic, echocardiographic, and laboratory parameters as compared to those with reduced exercise capacity. In contrast, patients with a peak VO_2_ below 20 mL/kg/min showed significantly lower lung volumes, including residual volume and total lung capacity, along with relevant differences in several CPET variables. In detail, a lower VO_2_ at anaerobic threshold (AT) was reached (11.6 ± 3.0 vs. 14.9 ± 2.6 mL/kg/min, *p* = 0.008), with a lower peak pulmonary ventilation (62.1 ± 13.0 vs. 77.5 ± 13.9 L/min, *p* = 0.006), potentially indicating a higher degree of deconditioning as compared to patients with a normal exercise capacity. Moreover, lower levels of total performance were reached (90.9 ± 24.4 vs. 129.8 ± 12.4 Watts, *p* > 0.001), with more circulatory limitations, as expressed by a lower peak heart rate and lower peak oxygen pulse. Of interest, patients with reduced exercise capacity showed signs of a lower ventilation–perfusion efficiency, with a dead space/tidal volume (Vd/Vt) reduction being observed in only 35.7% vs. 87.5% of patients with normal exercise capacity (*p* = 0.029). In keeping with this, we also observed a higher VE/VCO_2_ at AT (37.4 ± 4.7 vs. 31.5 ± 3.7, *p* = 0.003) and a trend toward a higher VE/VCO_2_ slope (36.3 ± 5.3 vs. 32.6 ± 4.5, *p* = 0.078) among participants with reduced exercise performance.

When considering parameters of vascular reactivity, a significantly higher FMD was documented in convalescent COVID-19 patients with a preserved as compared to those with a reduced exercise capacity (4.11% ± 2.08 vs. 2.54% ± 1.85, *p* = 0.048). In a multivariate analysis, after adjusting for gender, age, body mass index, hypertension, dyslipidemia, diabetes, and smoking habit, this result was substantially confirmed (β = 0.899, *p* = 0.038). No significant differences in BAD (*p* = 0.925), reactive hyperemia (*p* = 0.419), total share rate area under the curve (*p* = 0.641), and shear rate area under the curve from cuff deflation to peak diameter (*p* = 0.466) were found.

**Figure 1:**
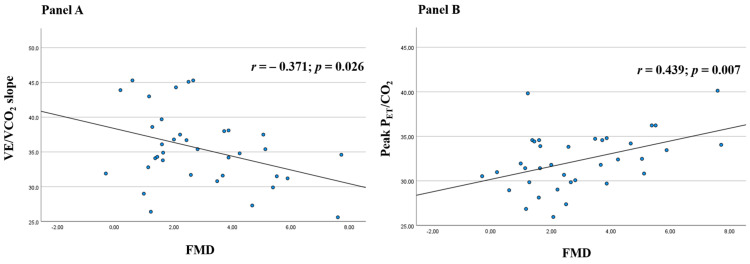
Scatter plots of Pearson’s correlations of flow-mediated dilation (FMD) with minute ventilation/carbon dioxide production (VE/VCO_2_) slope (**A**) and end-tidal carbon dioxide tension (P_ET_CO_2_) at peak (**B**) in convalescent coronavirus disease 2019 (COVID-19) patients.

In the overall study population, FMD values showed a significant Pearson’s correlation with two primary CPET parameters, namely VE/VCO_2_ slope (r = −0.371, *p* = 0.026, Figure 1A) and P_ET_CO_2_ at peak (r = 0.439, *p* = 0.007, Figure 1B), while no significant correlation was observed with other CPET variables both in parametric and in non-parametric tests. In multiple linear regressions, after adjusting for gender, age, body mass index, hypertension, dyslipidemia, diabetes, and smoking habit, FMD was the only independent predictor of VE/VCO_2_ slope (β = −1.308, *p* = 0.029) and peak P_ET_CO_2_ values (β = 0.779, *p* = 0.021). Finally, when stratifying our study population based on their ventilatory efficiency [16], fifteen patients with a ventilatory class III-IV (VE/VCO_2_ slope ≥ 36) exhibited significantly lower FMD values as compared to those (*n* = 21) with a ventilatory class I-II (2.18% ± 1.29 vs. 3.40% ± 2.26, *p* = 0.048).

## 4. Discussion

This pilot study represents the first attempt to explore the association between endothelial function and cardiopulmonary exercise performance in convalescent COVID-19 patients. In line with previous evidence [19,20,21], our findings confirmed that, after a severe-to-critical form of COVID-19, relevant functional limitations may persist and that these limitations may not only depend on physical deconditioning but also on a lower ventilation–perfusion efficiency. Moreover, results of our analyses suggest that the alteration of endothelial barrier properties in the systemic and pulmonary circulation may represent a key pathogenic mechanism of the reduced CPET performance.

Previous studies have already explored cardiopulmonary exercise capacity in COVID-19 survivors, trying to address the issue of whether the CPET limitations may depend on general deconditioning or parenchymal lung involvement. However, contrasting results have been reported, likely depending on the different inclusion and exclusion criteria among different studies. Rinaldo et al. suggested the absence of relevant functional sequelae on ventilatory and gas exchange response to exercise in COVID-19 survivors [5]. Accordingly, extrapulmonary factors were identified as the main reason for exercise limitation in one of the first reports on CPET in a small case-series of 10 moderate-to-severe COVID-19 survivors [22]. The key role of physical deconditioning was later confirmed in the largest study currently available on this issue, which reported only a one-third rate of reduced exercise capacity, likely due to a less severe disease in that patient group [6]. In our study, the analysis of the CPET parameters suggested a reduced exercise performance in almost 80% of COVID-19 survivors, who reported more circulatory limitations and worse ventilation–perfusion efficiency beyond a higher degree of physical deconditioning as compared to patients with normal peak VO_2_. It is important to highlight that these results come from a subset of severe-to-critical COVID-19 patients, with a median value of 12 out of 20 for the high-resolution CT total severity score. Therefore, in line with previous evidence [19,20,21], our findings may confirm the hypothesis that, in patients with a more severe disease course, the presence of CPET limitations may be reported in a relevant proportion of cases, depending on both physical deconditioning and interstitial lung involvement with impaired ventilation–perfusion efficiency. The fact that total lung capacity (TLC), forced vital capacity (FVC) and diffusion lung capacity for carbon monoxide (DLCO) were significantly lower in patients with reduced exercise performance further supports the potential influence of the residual restrictive pattern on the disabling manifestations and the possibility of recovery after the acute phase.

The other major finding of our study is the evidence of a higher FMD in patients with preserved, as compared to those with reduced, exercise capacity, confirmed in a multivariate analysis. FMD has been widely accepted as an accurate and non-invasive method for clinical assessment of endothelial function, providing important prognostic data beyond traditional cardiovascular risk factors [23]. The presence of an association between FMD values and two primary CPET parameters, namely VE/VCO_2_ slope and P_ET_CO_2_ at peak, suggests that the alteration of endothelial barrier properties in systemic and pulmonary circulation may be somehow related to the reduced ventilatory efficiency in COVID-19 patients, with our regression models and subgroup analyses supporting the possibility of a potential pathogenic role.

To date, it has been proven that SARS-CoV-2 is able to bind to the angiotensin-converting enzyme 2 (ACE2), normally expressed on human cells, helped by the transmembrane protease serine 2 (TMPRSS2) [24]. Therefore, it is reasonable to assume that human cells expressing ACE2 and TMPRSS2 on their surface represent SARS-CoV-2 target cells [25]. There is evidence that ECs show a large concentration of ACE2 on their surface, thus being a natural attack point for the virus [14]. Accordingly, SARS-CoV-2 has been isolated from ECs of various organs in COVID-19 patients, and a subsequent microvascular lymphocytic endotheliitis has been demonstrated [25]. However, the virus capacity to effectively infect ECs has recently been put into question [26]. Beyond the hypothesis of direct viral mechanisms, systemic inflammation plays an additional role in the disruption of endothelial barrier integrity in COVID-19, since inflammatory cytokines from activated leukocytes are able to bind to specific receptors on ECs, thus enhancing the expression of a number of mediators and adhesion molecules, including intercellular adhesion molecule-1 (ICAM-1), vascular cell adhesion molecule-1 (VCAM-1), and von Willebrand factor (vWF) [2,27]. This results in platelet activation and leukocyte adherence and extravasation [28], along with a decline in nitric oxide (NO) synthesis [29].

Overall, current evidence shows that endothelial damage due to direct or indirect viral action is associated with a procoagulant state and subsequent formation of microthrombi, resulting in multiorgan dysfunction and muscle damage in COVID-19 [30]. In keeping with this, endothelial dysfunction is associated to a lower NO bioavailability, with impaired smooth muscle cell relaxation and reduced vasodilation [14]. Overall, these pathological mechanisms may lead to a lower O_2_ supply to the periphery, thus contributing to acute sarcopenia and muscle weakness [31]. However, skeletal muscle involvement is only one aspect of deconditioning, which is a systemic adaptation to a less demanding environment. Endothelial dysfunction at a microcirculatory level can participate in cardiovascular, pulmonary, and autonomic dysfunction in COVID-19 patients, determining changes in energy metabolism and organ perfusion [32]. Conversely, it is important to highlight that, if acute physical inactivity is able to enhance basal shear-rate in large arteries [33], prolonged bed rest may decrease shear stress in the microcirculation, and a chronic decrease in shear stress is able to induce endothelial apoptosis and dysfunction [34].

Therefore, although our results consistently support the presence of an association between endothelial function and cardiopulmonary exercise performance in COVID-19, the nature of this association remains to be determined, since we cannot exclude that a dysfunctional endothelium may also be a consequence of inactivity rather than a pathogenic mechanism of the reduced CPET performance and lower ventilation–perfusion efficiency. Consequently, our preliminary findings deserve confirmation in preclinical studies and in robust clinical studies on a larger sample. Meanwhile, this interrelationship between endothelial barrier integrity and the functional limitations of the post-acute phase may suggest the usefulness of personalized strategies targeting and monitoring endothelial dysfunction [35,36,37], thus potentially contributing to reducing the increased cardiovascular risk of COVID-19 survivors [38]. A number of strategies directed against viral replication or systemic inflammation have been tested for COVID-19. Most of these strategies have the ability to act—at least in part—by reducing endothelial dysfunction and restoring the anticoagulant properties of the endothelium [39]. Among them, renin–angiotensin system (RAS) inhibitors and statins, which have shown to improve endothelial function in other clinical settings [40], have been tested in large observational studies and randomized trials with contrasting results in COVID-19 [41,42,43]. The positive impact of exercise on endothelial function has been known for a long time both in healthy subjects and in different clinical settings (e.g., chronic obstructive pulmonary disease, heart failure) [44,45,46,47]. A number of mechanisms have been proposed to explain the beneficial effects of exercise-based strategies on endothelial function, including mobilization of endothelial progenitor cells, upregulation of superoxide dismutase, and reduced uncoupling with increased phosphorylation of endothelial NO synthase [48]. In COVID-19, we previously suggested the potential beneficial effect of exercise-based rehabilitation on endothelial function [14]. However, larger studies with a controlled design and additional outcome measures are needed to clarify the possibility to restore endothelial integrity through personalized pharmacological and rehabilitation strategies.

Some potential limitations of our protocol need to be addressed. First, this pilot study was conducted on a relatively small sample, with a limited number of patients enrolled among those screened for eligibility. However, it is important to highlight that the high number of participants declared ineligible is due to the strict exclusion criteria, given the inclusion only of patients with no history of cardiovascular or pulmonary disease that could impact physical performance and vascular reactivity. Another relevant limitation of our observation is its gender-biased nature, as most of the patients included were male. We have previously demonstrated that no significant difference is observed in FMD values between convalescent COVID-19 patients and matched controls when specifically considering females, discussing the genetic and hormonal reasons potentially underlying this finding [24]. Therefore, our results cannot be generalized to both genders. The fact that the majority of COVID-19 patients admitted to our rehabilitation unit and, consequently, enrolled in our study were males substantially reflects the evidence of a disproportionately worse prognosis for male gender [49].

## 5. Conclusions

In line with previous evidence [2,50], our preliminary findings, although needing further confirmation, may suggest that endothelial dysfunction could be regarded as the common pathogenic background of most functional manifestations of COVID-19 during the acute and convalescent phases. Most importantly, our results confirm that endothelial dysfunction could be an additional and attractive therapeutic target in this clinical setting. Finally, we provided data consistent with the recommendation to periodically assess endothelial function during convalescence [3], in order to monitor the risk of long-term cardiovascular complications in COVID-19.

## Figures and Tables

**Table 1:** Main demographic and clinical characteristics of convalescent COVID-19 patients.

Variable	Overall	Normal Exercise Capacity	Reduced Exercise Capacity	*p* Value
	36	8	28	
**Demographic**				
Age, years	54.5 ± 10.6	54.9 ± 9.3	54.4 ± 11.1	0.918
Male gender, %	91.7	100	89.3	0.809
Active smokers, %	2.8	12.5	0	0.498
History of smoking, %	44.4	37.5	46.4	0.964
**Anthropometric**				
Weight, kg	85.0 (78.0–96.0)	82.5 (74.5–94.5)	85.0 (78.0–97.8)	0.421
BMI, kg/m^2^	27.9 (24.9–32.0)	25.3 (23.4–29.1)	28.1 (25.4–32.3)	0.077
**Acute phase COVID-19**				
Orotracheal intubation, %	2.8	0	3.6	1.000
NIMV/High-flow O_2_, %	61.1	50.0	64.3	0.749
Long-term O_2_ therapy, %	83.3	62.5	89.3	0.209
HRCT TSS (0–20)	12.0 (7.3–14.0)	9.0 (5.8–14.0)	12 (7.5–14.0)	0.513
**Pulmonary function tests**				
FEV_1_, L	2.8 ± 0.8	3.3 ± 0.5	2.7 ± 0.8	0.056
FEV_1_, % predicted	82.0 ± 20.6	92.8 ± 17.8	78.9 ± 20.6	0.095
FVC, L	3.4 ± 1.1	4.3 ± 0.9	3.2 ± 1.0	**0.008**
FVC, % predicted	79.9 ± 20.8	95.4 ± 17.6	75.5 ± 19.7	**0.015**
FEV_1_/FVC	82.4 ± 7.0	77.4 ± 8.0	83.8 ± 6.1	**0.019**
DLCO, mL/min/mmHg	19.1 ± 5.6	22.5 ± 5.1	18.0 ± 5.4	**0.045**
DLCO, % predicted	66.4 ± 18.3	74.6 ± 15.9	63.8 ± 18.5	0.073
DLCO/VA, mL/min/mmHg/L	3.9 ± 0.7	3.7 ± 0.5	3.9 ± 0.8	0.557
DLCO/VA, % predicted	90.1 ± 14.2	88.5 ± 9.7	90.5 ± 15.3	0.769
TLC, L	4.6 ± 1.4	5.9 ± 1.2	4.2 ± 1.3	**0.003**
TLC, % predicted	67.9 ± 18.3	82.0 ± 13.4	63.7 ± 17.7	**0.011**
RV, L	1.1 ± 0.7	1.5 ± 0.8	1.0 ± 0.7	0.092
RV, % predicted	49.3 ± 28.9	63.1 ± 29.3	45.2 ± 28.0	0.125
RV/TLC	24.4 ± 11.9	25.9 ± 9.5	24.0 ± 12.6	0.725
**Echocardiography**				
EF, %	57.9 ± 4.1	56.1 ± 4.5	58.4 ± 4.0	0.206
TAPSE, mm	25.2 ± 2.6	25.7 ± 5.2	23.4 ± 2.8	0.129
SPAP, mmHg	27.5 ± 5.9	25.7 ± 5.2	27.9 ± 6.0	0.380
TAPSE/SPAP, mm/mmHg	0.89 ± 0.19	1.01 ± 0.20	0.87 ± 0.18	0.085
E, cm/s	56.5 (46.3–66.3)	64.5 (50.2–71.8)	55.0 (44.8–64.5)	0.145
A, cm/s	63.0 ± 11.4	60.0 ± 15.6	63.6 ± 10.6	0.490
E/A ratio	0.86 (0.73–1.15)	1.09 (0.82–1.33)	0.83 (0.72–1.12)	0.110
E/E’ ratio	5.0 (4.0–6.0)	4.0 (3.5–5.5)	5.0 (4.0–6.0)	0.190
DT, ms	185.8 ± 50.8	178.5 ± 44.4	187.4 ± 52.7	0.703
**Blood laboratory parameters**				
Hemoglobin, g/dL	13.0 ± 1.5	13.5 ± 0.9	12.9 ± 1.6	0.294
Creatinine, mg/dL	0.82 ± 0.14	0.91 ± 0.14	0.81 ± 0.13	0.069
GFR, mL/min/1.73 m^2^	98.8 ± 15.1	94.4 ± 12.9	100.1 ± 15.7	0.359
BUN, mg/dL	35.2 ± 11.1	38.6 ± 12.3	34.5 ± 11.0	0.461
AST, UI/L	19.5 (15.0–34.0)	22.0 (14.3–29.3)	19.0 (15.8–35.3)	0.796
ALT, UI/L	56.0 (30.0–86.3)	61.5 (27.0–97.0)	55.0 (33.0–80.3)	0.796
γGT, UI/L	38.0 (30.0–54.8)	36.0 (27.0–54.0)	40.0 (32.0–63.0)	0.592
Procalcitonin, ng/mL	0.03 (0.02–0.05)	0.02 (0.01–0.03)	0.03 (0.02–0.06)	0.115
CRP, mg/dL	2.6 (1.1–7.3)	1.5 (0.6–7.0)	3.2 (1.2–8.1)	0.275
D-Dimer, ng/mL	290.0 (270.0–490.0)	270.0 (250.0–342.5)	310.0 (270.0–320.0)	0.105
Troponin I, pg/mL	3.0 (2.0–5.0)	2.5 (2.0–3.3)	4.0 (2.0–5.5)	0.190
Myoglobin, µg/L	34.0 (27.5–53.0)	36.5 (27.5–50.0)	33.0 (27.0–54.0)	0.854
CK-MB, ng/mL	0.85 ± 0.53	1.10 ± 0.79	0.77 ± 0.44	0.367
CPK, UI/L	29.0 (20.0–42.0)	29.0 (25.0–45.0)	30.5 (20.0–41.3)	0.872
BNP, pg/mL	11.8 (10.0–30.9)	10.6 (10.0–16.5)	15.2 (10.0–32.0)	0.473
**Blood pressure**				
24 h SBP, mmHg	121.9 ± 12.8	115.5 ± 7.5	123.9 ± 13.6	0.176
24 h DBP, mmHg	80.0 ± 6.3	78.3 ± 6.9	80.6 ± 6.3	0.457
**Comorbidities**				
Hypertension, %	50.0	37.5	53.6	0.688
Diabetes mellitus, %	11.1	12.5	10.7	1.000
Dyslipidemia, %	11.1	25.0	7.1	0.436
Obesity, %	30.6	12.5	35.7	0.411
OSAS, %	8.3	12.5	7.1	1.000
**Pharmacological therapy**				
Statins, %	12.1	28.6	7.7	0.395
β-blockers, %	15.2	0	19.2	0.506
ACE-I, %	21.2	26.8	19.2	0.987
CCB, %	15.6	0	19.2	0.585
ARB, %	18.2	0	23.1	0.394
**COVID-19 therapy**				
Corticosteroids, %	70.6	57.1	74.1	0.681
Antibiotics, %	23.5	14.3	25.9	0.883
LMWH, %	67.6	57.1	70.4	0.831
**CPET—** **Performance**				
Peak VO_2_, mL/kg/min	16.6 ± 3.9	21.7 ± 1.9	15.1 ± 3.0	**<0.001**
Peak VO_2_, % predicted	62.4 ± 16.1	75.9 ± 16.3	58.6 ± 14.1	**0.006**
Work, Watt	99.6 ± 27.5	129.8 ± 12.4	90.9 ± 24.4	**<0.001**
Borg dyspnea (0–10)	3.0 (3.0–5.0)	3.0 (3.0–5.0)	3.0 (3.0–5.0)	0.723
**CPET—** **Ventilation**				
Peak VE, L/min	65.5 ± 14.6	77.5 ± 13.9	62.1 ± 13.0	**0.006**
Peak VE, % predicted	67.6 ± 14.4	76.3 ± 15.9	65.1 ± 13.2	0.052
Breathing reserve, %	29.8 ± 17.0	32.9 ± 12.0	28.9 ± 18.3	0.596
**CPET—** **Circulation**				
Peak HR, beats/min	147.0 (124.5–158.8)	159.0 (155.3–160.0)	142.0 (123.3–154.8)	**0.030**
Peak HR, % predicted	86.2 ± 11.8	94.3 ± 11.4	83.9 ± 11.1	**0.027**
HRR at 1 min, beats	19.0 ± 9.5	18.8 ± 7.3	19.0 ± 10.2	0.942
Peak O_2_ pulse, L/stroke	9.9 (8.3–11.7)	11.3 (10.1–13.7)	9.6 (8.3–10.9)	**0.044**
Peak O_2_ pulse, % predicted	69.0 (60.0–90.5)	82.0 (69.0–91.8)	65.5 (57.5–82.5)	0.135
**CPET—** **Gas exchange**				
VE/VCO_2_ slope	35.5 ± 5.3	32.6 ± 4.5	36.3 ± 5.3	0.078
VE/VCO_2_ at AT	36.1 ± 5.1	31.5 ± 3.7	37.4 ± 4.7	**0.003**
Peak RER	1.19 (1.13–1.27)	1.18 (1.14–1.30)	1.20 (1.13–1.30)	0.780
Peak P_ET_CO_2_, mmHg	32.3 ± 3.2	33.8 ± 3.6	31.8 ± 3.0	0.126
Vd/Vt reduction, %	47.2	87.5	35.7	**0.029**
**CPET—** **Anaerobic threshold**				
AT, mL/kg/min	12.4 ± 3.2	14.9 ± 2.6	11.6 ± 3.0	**0.008**
VO_2_/Work slope	9.2 ± 1.3	9.7 ± 0.9	9.1 ± 1.3	0.220
**Vascular reactivity**				
FMD, %	2.89 ± 1.99	4.11 ± 2.08	2.54 ± 1.85	**0.048**
BAD, mm	4.15 ± 0.65	4.17 ± 0.44	4.14 ± 0.70	0.925
RH	1.86 ± 0.15	1.22 ± 0.15	1.17 ± 0.15	0.419
SR_AUC-TOT_	52,157.4 (32,883.1–66,629.7)	54,343.3 (34,142.4–70,067.6)	50,869.3 (32,883.1–6629.7)	0.641
SR_AUC_	19,702.5 (11,114.9–31,746.6)	23,565.3 (10,142.8–35,303.7)	18,800.0 (11,114.9–31,746.6)	0.466

COVID-19: coronavirus disease 2019; BMI: body mass index; NIMV: non-invasive mechanical ventilation; O_2_: oxygen; HRCT TSS: high-resolution computed tomography total severity score; FEV_1_: forced expiratory volume in 1 s; FVC: forced vital capacity; DLCO: diffusion lung of carbon monoxide; VA: alveolar volume; TLC: total lung capacity; RV: residual volume; EF: ejection fraction; TAPSE: tricuspid annular plane systolic excursion; SPAP: systolic pulmonary artery pressure; E: early diastolic flow velocity; A: late diastolic flow velocity; E/E’: early diastolic flow velocity/lateral E′ velocity; DT: deceleration time; GFR: glomerular filtration rate; BUN: blood urea nitrogen; AST: aspartate aminotransferase; ALT: alanine aminotransferase; γGT: gamma-glutamyl transferase; CRP: C-reactive protein; CK-MB: creatine kinase-MB; CPK: creatine phosphokinase; BNP: brain natriuretic peptide; SBP: systolic blood pressure; DBP: diastolic blood pressure; OSAS: obstructive sleep apnea syndrome; ACE-I: angiotensin-converting enzyme inhibitors; CCB: calcium channel blockers; ARB: angiotensin II receptor blockers; LMWH: low molecular weight heparin; CPET: cardiopulmonary exercise test; VO_2_: oxygen uptake; Borg dyspnea: Borg scale of dyspnea at peak; VE: pulmonary ventilation; HR: heart rate; HRR: heart rate recovery; VE/VCO_2_: minute ventilation/carbon dioxide production; AT: anaerobic threshold; RER: respiratory exchange ratio; P_ET_CO_2_: end-tidal carbon dioxide tension; Vd/Vt: dead space/tidal volume; FMD: flow-mediated dilation; BAD: brachial artery diameter; RH: reactive hyperemia; SR_AUC-TOT_: total share rate area under the curve; SR_AUC_: shear rate area under the curve from cuff deflation to peak diameter. Continuous data are presented as mean ± standard deviation or median (1st–3rd quartile) in case of skewed distribution. Categorical variables are summarized as relative frequencies. A *p* value < 0.05 was considered statistically significant (bold font).

## Data Availability

The data supporting the findings of this study are available from the corresponding authors upon reasonable request.

## Supplementary Materials

The following supporting information can be downloaded at: https://www.mdpi.com/article/10.3390/jcm11051452/s1, Supplemental Figure S1: Flow chart of study participants.

## References

[1] Vane J.R., Anggard E.E., Botting R.M. (1990). Regulatory functions of the vascular endothelium. N. Engl. J. Med..

[2] Calabretta E., Moraleda J.M., Iacobelli M., Jara R., Vlodavsky I., O’Gorman P., Pagliuca A., Mo C., Baron R.M., Aghemo A. (2021). COVID-19-induced endotheliitis: Emerging evidence and possible therapeutic strategies. Br. J. Haematol..

[3] Evans P.C., Rainger G.E., Mason J.C., Guzik T.J., Osto E., Stamataki Z., Neil D., Hoefer I.E., Fragiadaki M., Waltenberger J. (2020). Endothelial dysfunction in COVID-19: A position paper of the ESC Working Group for Atherosclerosis and Vascular Biology, and the ESC Council of Basic Cardiovascular Science. Cardiovasc. Res..

[4] Thijssen D.H.J., Bruno R.M., Van Mil A.C.C.M., Holder S.M., Faita F., Greyling A., Zock P.L., Taddei S., Deanfield J.E., Luscher T. (2019). Expert consensus and evidence-based recommendations for the assessment of flow-mediated dilation in humans. Eur. Heart J..

[5] Rinaldo R.F., Mondoni M., Parazzini E.M., Pitari F., Brambilla E., Luraschi S., Balbi M., Papa G.F.S., Sotgiu G., Guazzi M. (2021). Deconditioning as main mechanism of impaired exercise response in COVID-19 survivors. Eur. Respir. J..

[6] Skjørten I., Ankerstjerne O.A.W., Trebinjac D., Brønstad E., Rasch-Halvorsen Ø., Einvik G., Lerum T.V., Stavem K., Edvardsen A., Ingul C.B. (2021). Cardiopulmonary exercise capacity and limitations 3 months after COVID-19 hospitalisation. Eur. Respir. J..

[7] Bai C., Chotirmall S.H., Rello J., Alba G.A., Ginns L.C., Krishnan J.A., Rogers R., Bendstrup E., Burgel P.-R., Chalmers J.D. (2020). Updated guidance on the management of COVID-19: From an American Thoracic Society/European Respiratory Society coordinated International Task Force (29 July 2020). Eur. Respir. Rev..

[8] World Health Organization (2021). WHO COVID-19 Clinical Management: Living Guidance. https://apps.who.int/iris/handle/10665/338882.

[9] Von Elm E., Altman D.G., Egger M., Pocock S.J., Gøtzsche P.C., Vandenbroucke J.P. (2007). The Strengthening the Reporting of Observational Studies in Epidemiology (STROBE) statement: Guidelines for reporting observational studies. Lancet.

[10] Miller M.R., Hankinson J., Brusasco V., Burgos F., Casaburi R., Coates A., Crapo R., Enright P., Van Der Grinten C.P.M., Gustafsson P. (2005). Standardisation of spirometry. Eur. Respir. J..

[11] Wanger J., Clausen J.L., Coates A., Pedersen O.F., Brusasco V., Burgos F., Casaburi R., Crapo R., Enright P., Van Der Grinten C.P.M. (2005). Standardisation of the measurement of lung volumes. Eur. Respir. J..

[12] MacIntyre N., Crapo R.O., Viegi G., Johnson D.C., Van Der Grinten C.P.M., Brusasco V., Burgos F., Casaburi R., Coates A., Enright P. (2005). Standardisation of the single-breath determination of carbon monoxide uptake in the lung. Eur. Respir. J..

[13] Corretti M.C., Anderson T.J., Benjamin E., Celermajer D., Charbonneau F., Creager M.A., Deanfield J., Drexler H., Gerhard-Herman M., Herrington D. (2002). Guidelines for the ultrasound assessment of endothelial-dependent flow-mediated vasodilation of the brachial artery: A report of the International Brachial Artery Reactivity Task Force. J. Am. Coll. Cardiol..

[14] Ambrosino P., Molino A., Calcaterra I., Formisano R., Stufano S., Spedicato G., Motta A., Papa A., Di Minno M., Maniscalco M. (2021). Clinical Assessment of Endothelial Function in Convalescent COVID-19 Patients Undergoing Multidisciplinary Pulmonary Rehabilitation. Biomedicines.

[15] Weisman I.M., Marciniuk D., Martinez F.J., Sciurba F., Sue D., Myers J., Casaburi R., Marciniuk D., Beck K., Zeballos J. (2003). ATS/ACCP statement on cardiopulmonary exercise testing. Am. J. Respir. Crit. Care Med..

[16] Guazzi M., Arena R., Halle M., Piepoli M.F., Myers J., Lavie C.J. (2018). 2016 focused update: Clinical recommendations for cardiopulmonary exercise testing data assessment in specific patient populations. Eur. Heart J..

[17] Weber K.T., Janicki J.S., McElroy P.A. (1987). Determination of aerobic capacity and the severity of chronic cardiac and circulatory failure. Circulation.

[18] Radtke T., Vogiatzis I., Urquhart D.S., Laveneziana P., Casaburi R., Hebestreit H. (2019). Standardization of cardiopulmonary exercise testing in chronic lung diseases: Summary of key findings from the ERS task force. Eur. Respir. J..

[19] Dorelli G., Braggio M., Gabbiani D., Busti F., Caminati M., Senna G., Girelli D., Laveneziana P., Ferrari M., Sartori G. (2021). Importance of Cardiopulmonary Exercise Testing amongst Subjects Recovering from COVID-19. Diagnostics.

[20] Mancini D.M., Brunjes D.L., Lala A., Trivieri M.G., Contreras J.P., Natelson B.H. (2021). Use of Cardiopulmonary Stress Testing for Patients With Unexplained Dyspnea Post-Coronavirus Disease. JACC Heart Fail.

[21] Crisafulli E., Dorelli G., Sartori G., Dalle Carbonare L. (2021). Exercise ventilatory inefficiency may be a relevant CPET-feature in COVID-19 survivors. Int. J. Cardiol..

[22] Gao Y., Chen R., Geng Q., Mo X., Zhan C., Jian W., Li S., Zheng J. (2021). Cardiopulmonary exercise testing might be helpful for interpretation of impaired pulmonary function in recovered COVID-19 patients. Eur. Respir. J..

[23] Ambrosino P., Lupoli R., Iervolino S., De Felice A., Pappone N., Storino A., Di Minno M.N.D. (2017). Clinical assessment of endothelial function in patients with chronic obstructive pulmonary disease: A systematic review with meta-analysis. Intern. Emerg. Med..

[24] Ambrosino P., Calcaterra I., Molino A., Moretta P., Lupoli R., Spedicato G., Papa A., Motta A., Maniscalco M., Di Minno M. (2021). Persistent Endothelial Dysfunction in Post-Acute COVID-19 Syndrome: A Case-Control Study. Biomedicines.

[25] Varga Z., Flammer A.J., Steiger P., Haberecker M., Andermatt R., Zinkernagel A.S., Mehra M.R., Schuepbach R.A., Ruschitzka F., Moch H. (2020). Endothelial cell infection and endotheliitis in COVID-19. Lancet.

[26] McCracken I.R., Saginc G., He L., Huseynov A., Daniels A., Fletcher S., Peghaire C., Kalna V., Andaloussi-Mae M., Muhl L. (2021). Lack of Evidence of Angiotensin-Converting Enzyme 2 Expression and Replicative Infection by SARS-CoV-2 in Human Endothelial Cells. Circulation.

[27] Matsuishi Y., Mathis B., Shimojo N., Subrina J., Okubo N., Inoue Y. (2021). Severe COVID-19 Infection Associated with Endothelial Dysfunction Induces Multiple Organ Dysfunction: A Review of Therapeutic Interventions. Biomedicines.

[28] Hottz E.D., Azevedo-Quintanilha I.G., Palhinha L., Teixeira L., Barreto E.A., Pao C.R.R., Righy C., Franco S., Souza T.M.L., Kurtz P. (2020). Platelet activation and platelet-monocyte aggregate formation trigger tissue factor expression in patients with severe COVID-19. Blood.

[29] Green S.J. (2020). Covid-19 accelerates endothelial dysfunction and nitric oxide deficiency. Microbes Infect..

[30] Ruhl L., Pink I., Kühne J.F., Beushausen K., Keil J., Christoph S., Sauer A., Boblitz L., Schmidt J., David S. (2021). Endothelial dysfunction contributes to severe COVID-19 in combination with dysregulated lymphocyte responses and cytokine networks. Signal Transduct. Target Ther..

[31] Piotrowicz K., Gasowski J., Michel J.P., Veronese N. (2021). Post-COVID-19 acute sarcopenia: Physiopathology and management. Aging Clin. Exp. Res..

[32] Coupé M., Fortrat J.O., Larina I., Gauquelin-Koch G., Gharib C., Custaud M.A. (2009). Cardiovascular deconditioning: From autonomic nervous system to microvascular dysfunctions. Respir. Physiol. Neurobiol..

[33] Thijssen D.H.J., Maiorana A.J., O’Driscoll G., Cable N.T., Hopman M.T.E., Green D.J. (2010). Impact of inactivity and exercise on the vasculature in humans. Eur. J. Appl. Physiol..

[34] Demiot C., Dignat-George F., Fortrat J.O., Sabatier F., Gharib C., Larina I., Gauquelin-Koch G., Hughson R., Custaud M.A. (2007). WISE 2005: Chronic bed rest impairs microcirculatory endothelium in women. Am. J. Physiol. Heart Circ. Physiol..

[35] Lanza G.A., Golino M., Villano A., Lanza O., Lamendola P., Fusco A., Leggio M. (2020). Cardiac Rehabilitation and Endothelial Function. J. Clin. Med..

[36] Guo Y., Ledesma R.A., Peng R., Liu Q., Xu D. (2017). The Beneficial Effects of Cardiac Rehabilitation on the Function and Levels of Endothelial Progenitor Cells. Heart Lung Circ..

[37] Ambrosino P., Papa A., Maniscalco M., Di Minno M.N.D. (2021). COVID-19 and functional disability: Current insights and rehabilitation strategies. Postgrad. Med. J..

[38] Tu T.M., Seet C.Y.H., Koh J.S., Tham C.H., Chiew H.J., De Leon J.A., Chua C.Y.K., Hui A.C., Tan S.S.Y., Vasoo S.S. (2021). Acute Ischemic Stroke During the Convalescent Phase of Asymptomatic COVID-2019 Infection in Men. JAMA Netw. Open.

[39] Deng H., Tang T.X., Chen D., Tang L.S., Yang X.P., Tang Z.H. (2021). Endothelial Dysfunction and SARS-CoV-2 Infection: Association and Therapeutic Strategies. Pathogens.

[40] Shahin Y., Khan J.A., Samuel N., Chetter I. (2011). Angiotensin converting enzyme inhibitors effect on endothelial dysfunction: A meta-analysis of randomised controlled trials. Atherosclerosis.

[41] Hippisley-Cox J., Young D., Coupland C., Channon K.M., Tan P.S., Harrison D.A., Rowan K., Aveyard P., Pavord I.D., Watkinson P.J. (2020). Risk of severe COVID-19 disease with ACE inhibitors and angiotensin receptor blockers: Cohort study including 8.3 million people. Heart.

[42] Lopes R.D., Macedo A.V.S., de Barros E.S.P.G.M., Moll-Bernardes R.J., Dos Santos T.M., Mazza L., Feldman A., D’Andrea Saba Arruda G., de Albuquerque D.C., Camiletti A.S. (2021). Effect of Discontinuing vs Continuing Angiotensin-Converting Enzyme Inhibitors and Angiotensin II Receptor Blockers on Days Alive and Out of the Hospital in Patients Admitted With COVID-19: A Randomized Clinical Trial. JAMA.

[43] Catanzaro M., Fagiani F., Racchi M., Corsini E., Govoni S., Lanni C. (2020). Immune response in COVID-19: Addressing a pharmacological challenge by targeting pathways triggered by SARS-CoV-2. Signal Transduct. Target Ther..

[44] Sinoway L.I., Musch T.I., Minotti J.R., Zelis R. (1986). Enhanced maximal metabolic vasodilatation in the dominant forearms of tennis players. J. Appl. Physiol..

[45] Merlo C., Bernardi E., Bellotti F., Pomidori L., Cogo A. (2020). Supervised exercise training improves endothelial function in COPD patients: A method to reduce cardiovascular risk?. ERJ Open Res..

[46] Kitzman D.W., Brubaker P.H., Herrington D.M., Morgan T.M., Stewart K.P., Hundley W.G., Abdelhamed A., Haykowsky M.J. (2013). Effect of endurance exercise training on endothelial function and arterial stiffness in older patients with heart failure and preserved ejection fraction: A randomized, controlled, single-blind trial. J. Am. Coll. Cardiol..

[47] Dai R., Zhuo H., Chen Y., Zhang K., Dong Y., Chen C., Wang W. (2021). Mechanism of Isosorbide Dinitrate Combined with Exercise Training Rehabilitation to Mobilize Endothelial Progenitor Cells in Patients with Coronary Heart Disease. Bioengineered.

[48] Ross M.D., Malone E., Florida-James G. (2016). Vascular Ageing and Exercise: Focus on Cellular Reparative Processes. Oxid. Med. Cell. Longev..

[49] Grasselli G., Zangrillo A., Zanella A., Antonelli M., Cabrini L., Castelli A., Cereda D., Coluccello A., Foti G., Fumagalli R. (2020). Baseline characteristics and outcomes of 1591 patients infected with SARS-CoV-2 admitted to ICUs of the Lombardy Region, Italy. JAMA.

[50] Bonaventura A., Vecchié A., Dagna L., Martinod K., Dixon D.L., Van Tassell B.W., Dentali F., Montecucco F., Massberg S., Levi M. (2021). Endothelial dysfunction and immunothrombosis as key pathogenic mechanisms in COVID-19. Nat. Rev. Immunol..

