# Supplementary material for: Cardiopulmonary Exercise Performance and Endothelial Function in Convalescent COVID-19 Patients
Source: J Clin Med. 2022 Mar 7;11(5):1452. doi: 10.3390/jcm11051452 (PMC8911200; doi:10.3390/jcm11051452)
Supplement: Supplementary file 1 [file jcm-11-01452-s001.zip › jcm-1610042-supplementary.pdf]

Supplementary Material

Article

Cardiopulmonary Exercise Performance and Endothelial Function in Convalescent COVID-19 Patients

Pasquale Ambrosino [1,*,†], Paolo Parrella [2,†], Roberto Formisano [1], Giovanni Perrotta [1], Silvestro Ennio D'Anna [3], Marco Mosella [3], Antimo Papa [1,‡] and Mauro Maniscalco [3,*,‡]

1 Istituti Clinici Scientifici Maugeri IRCCS, Cardiac Rehabilitation Unit of Telese Terme Institute, 82037 Telese Terme, Italy; roberto.formisano@icsmaugeri.it (R.F.); giovanni.perrotta@icsmaugeri.it (G.P.); antimo.papa@icsmaugeri.it (A.P.)
2 Ospedale Sacro Cuore di Gesù Fatebenefratelli, 82100 Benevento, Italy; paoloparrella01@libero.it
3 Istituti Clinici Scientifici Maugeri IRCCS, Pulmonary Rehabilitation Unit of Telese Terme Institute,
82037 Telese Terme, Italy; silvestro.danna@icsmaugeri.it (S.E.D.); marco.mosella@icsmaugeri.it (M.M.)
* Correspondence: pasquale.ambrosino@icsmaugeri.it (P.A.); mauro.maniscalco@icsmaugeri.it (M.M.)
† These authors contributed equally to this work.
‡ These authors contributed equally to this work.

Table of contents:

**Supplemental Figure 1**	**Flow chart of study participants.**

Figure S1. Flow chart of study participants.

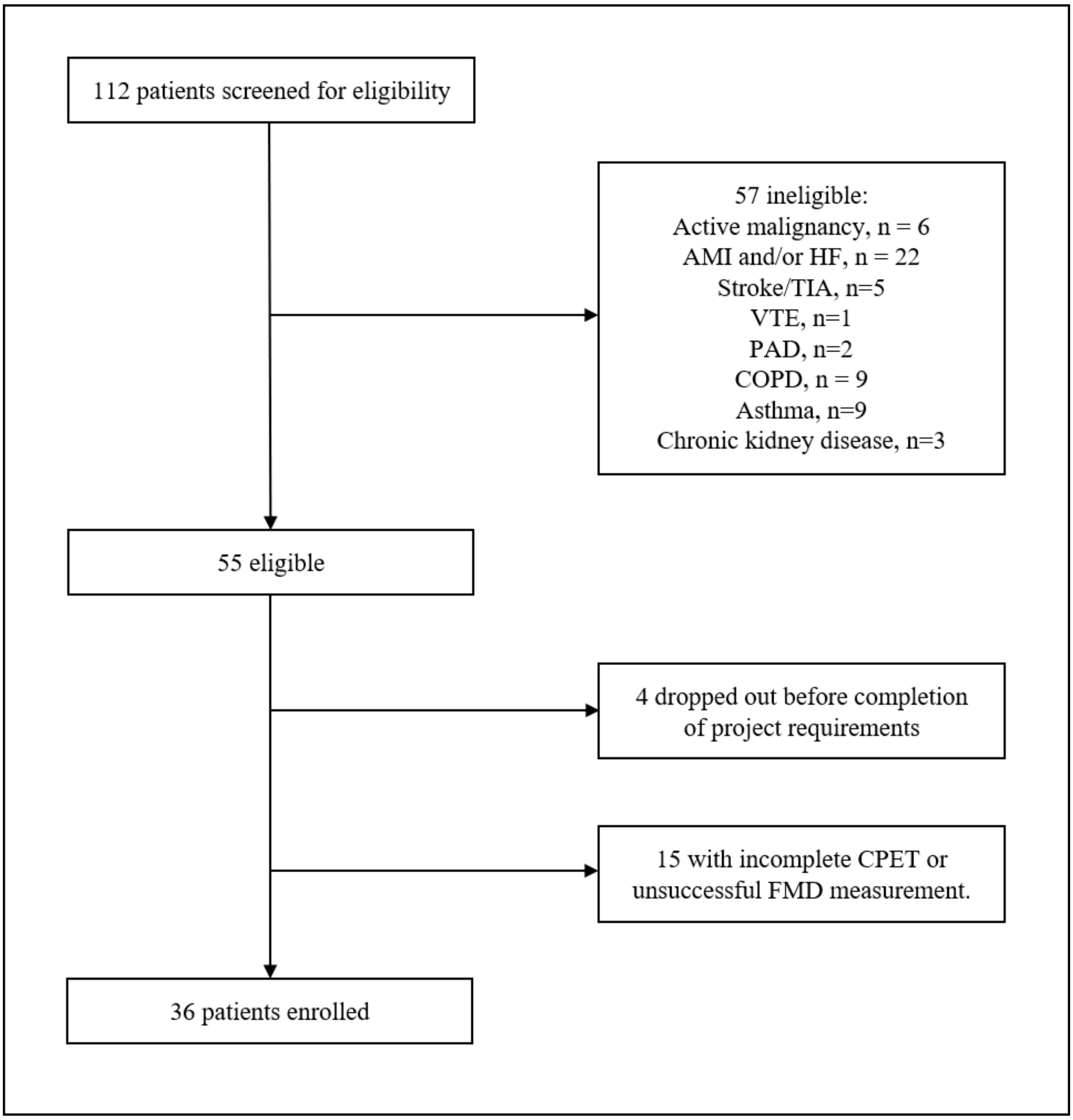

AMI: acute myocardial infarction; HF: heart failure; TIA: transient ischaemic attack; VTE: venous thromboembolism; PAD: peripheral artery disease; COPD: chronic obstructive pulmonary disease; CPET: cardiopulmonary exercise testing; FMD: flow-mediated dilation.
